# Simple, Rapid and Validated LC Determination of Lopinavir in Rat Plasma and its Application in Pharmacokinetic Studies

**DOI:** 10.3797/scipharm.1107-24

**Published:** 2011-09-17

**Authors:** Rahul Vats, Aditya Narasimha Murthy, Punna Rao Ravi

**Affiliations:** BITS-Pilani Hyderabad Campus, Jawaharnagar, Ranga Reddy (Dist.), Andhra Pradesh, India

**Keywords:** Liquid chromatography, Lopinavir, HIV-1 protease inhibitor, Rat plasma, HPLC, Pharmacokinetic studies

## Abstract

Lopinavir is a new specific and potent HIV-1 protease inhibitor. A simple and rapid Reverse Phase High-Performance Liquid Chromatographic method using UV detection was developed and validated for the analysis of lopinavir in rat plasma under isocratic conditions. The method involves a single step protein precipitation technique. The detector response was linear over the concentration range of 250 to 4000 ng mL ^−1^. High recovery ranging from 97.5 to 101.2 percent was obtained which precludes the use of internal standard. The developed method was validated as per standard guidelines. Validation of the developed method demonstrated accuracy, precision and selectivity of the proposed method. The drug was found to be stable under various processing and storage conditions. This rapid and cost-effective method was successfully applied in the estimation of lopinavir and determination of various pharmacokinetic parameters during post intravenous bolus administration of the drug in rats. The developed method can be suitably employed in preclinical pharmacokinetic evaluation of new formulations designed to improve the bioavailability of lopinavir.

## Introduction

Lopinavir (LPV) is a potent HIV protease inhibitor (PI) and a key ingredient of Highly Active Anti-Retroviral Therapy (HAART) [1]. LPV was developed by Abbott Laboratories to improve pharmacokinetics and to reduce HIV resistance of the company’s earlier protease inhibitor, Ritonavir (RTV) [2]. LPV has low oral bioavailability when administered alone because of poor solubility, high first pass metabolism [3] and P-gp efflux [4]. RTV is co-administered with LPV orally in HAART in order to improve the bioavailability of LPV. RTV increases bioavailability of LPV due to its inhibitory effects on gut and liver Cytochrome (CYP) P450 enzymes and permeability glycoprotein (P-gp) efflux system [5].

**Fig. 1:**
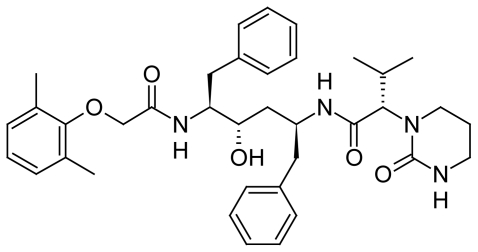
Chemical structure of LPV

LPV (Fig. 1) is chemically designated as (2*S*)-*N*-[(1*S*,3*S*,4*S*)-1-benzyl-4-{[(2,6-dimethyl-phenoxy)acetyl]amino}-3-hydroxy-5-phenylpentyl]-3-methyl-2-(2-oxotetrahydropyrimidin-1(2*H*)-yl)butanamide. Its molecular formula is C_37_H_48_N_4_O_5_ and its molecular weight is 628.80 [6].

Several research groups have been working on the development of novel delivery systems containing LPV alone in the effective treatment of HIV/AIDS. Such delivery systems will avoid heavy pill burden of LPV and RTV co-formulation, and improve patient compliance and adherence to therapy which are very vital for treatment against HIV/AIDS. In line with this, researchers have tried to improve solubility and bioavailability of LPV using microparticulate and nanocarrier systems [7, 8]. Agarwal and co-workers have tried prodrug approach for LPV [9]. Outcome of improving bioavailability of LPV using novel drug delivery systems containing LPV alone have been positive in preclinical studies, conducted on rats and mice [10]. Such research endeavors need a simple, rapid and cost-effective bioanalytical method to quantify the LPV concentration in rat plasma.

Analytical methods for PIs like amprenavir, indinavir, nelfinavir, ritonavir and saquinavir have been reported in human plasma and/or other biological matrices [11–21]. Simultaneous methods for estimation of LPV in combination with various PIs in human plasma, mainly by liquid chromatography tandem mass spectrometry (LC-MS), have been reported [22–29]. High-Performance Liquid Chromatographic (HPLC) methods with UV detection have also been explored successfully for simultaneous determination of LPV with other PIs using isocratic as well as gradient elution techniques [30, 31]. A method for estimation of LPV alone in human plasma matrix using HPLC system has been validated [32].

Different sample preparation techniques like protein precipitation, liquid-liquid extraction [23–25] and solid phase extraction [22] have been explored for analysis of various PIs. Among the reported techniques, protein precipitation technique is considered to be a simple, fast and cost-effective technique for extraction of drug from plasma matrix [28].

Although bioanalytical methods reported for estimation of LPV are found to be very sensitive, precise and accurate, they use costly LC-MS techniques and involve tedious and time consuming sample preparation steps. Extensive literature survey did not reveal any bioanalytical method for estimation of LPV in rat plasma which can be useful for *in vivo* pharmacokinetic evaluation of drug delivery systems containing LPV alone in rat models. Therefore it was envisaged to develop a simple, rapid, sensitive, accurate and reliable HPLC method for estimation of LPV in rat plasma.

This paper deals with development and validation of a HPLC method, with UV detection, for determination of LPV in rat plasma using protein precipitation technique. The developed HPLC method was used in the quantification of LPV in plasma samples obtained from *in vivo* pharmacokinetic studies in rats.

## Experimental

### Chemicals and Reagents

LPV was obtained as gift sample from Matrix Laboratories, India. Acetonitrile (HPLC grade), methanol (HPLC grade) and ammonium acetate (LiChropur^®^) were purchased from Merck laboratories, India. Ethanol and polyethylene glycol (PEG) 400 were purchased from S.D. Fine Chem. Ltd., India. Milli-Q water purification system (Millipore, USA) was used for obtaining high quality HPLC grade water.

### Instruments

The liquid chromatography system employed was Shimadzu HPLC (Shimadzu, Japan) with solvent delivery system of two pumps (Model LC-20AD, Prominence Liquid Chromatograph, Shimadzu, Japan), an auto injector (Model SIL-20A HT, Prominence Auto Sampler, Shimadzu, Japan) and photo diode array (PDA) UV detector (Model SPD-M20A, Prominence Diode Array Detector, Shimadzu, Japan). Data collection and integration was accomplished using LC Solutions, 1.25 version software.

Other instruments used in the method development and validation include vortex mixer (Model VX-200, Labnet International Inc., USA), sonicator (Model SONICA^®^ 2200 MH, Soltec, Italy), refrigerated centrifuge (Model C-24 BL, Remi, India) and deep freezer (Model BFS-345-S, Celfrost Innovations Pvt. Ltd., India). pH meter (Model pHTestr 30, Eutech Instruments, Singapore) was used for measuring pH of all buffer systems. Membrane filters of 0.22 μm (Millipore, USA) were used for filtration of aqueous phase used in the mobile phase system.

### Chromatographic Conditions

An endcapped C18 reverse phase (RP) column (Luna^®^, 250 mm long and 4.6 mm internal diameter, particle size 5 μm, Phenomenex, USA) equipped with a guard column of same packing material was used for the study. The isocratic mobile phase consisted of an aqueous phase (10 mM ammonium acetate, pH 6.5) and acetonitrile (35:65 *v/v*). Buffer was filtered through 0.22 μm Millipore membrane filter. The HPLC system was stabilized for 1 h at 1 mL min ^−1^ flow rate, through baseline monitoring prior to actual analysis. LPV was monitored at wavelength of 210 nm. An injection volume of 100 μL was optimized for final method.

### Collection of Blood and Separation of Plasma

Blood was collected from retro-orbital plexus of Wistar rats (Raj Biotech, India) weighing between 180 to 220 g. Prior permission was obtained for all experiments involving animals from the Institutional Animal Ethics Committee. Clear supernatant plasma was separated from blood after the centrifugation at 3400 rpm, 4° C, for 10 min. Samples were kept at −20 °C till further analysis.

### Method Development

In the process of analytical method development for LPV, mobile phase composition and flow rate were optimized by trying different aqueous phase and non-aqueous phase combinations at different flow rates. Various buffers with different pH and in varying compositions with acetonitrile and/or methanol were investigated.

Mobile phase composition and flow rate were finally selected based on the criteria of peak properties (retention time and asymmetric factor), sensitivity (height and area), ease of preparation and applicability of the method for *in vivo* studies in rats.

### Calibration Curve

Primary stock solution of LPV (1 mg mL ^−1^) was prepared in volumetric flask by dissolving accurately weighed amount of LPV in methanol. Secondary stock solutions of LPV, analytical standards for studying the absolute recovery of plasma standards (250, 500, 1000, 1500, 2000, 2500, 3000, 4000 ng mL ^−1^) and analytical quality control samples for studying the absolute recovery of plasma quality control samples (800, 1600, 3200 ng mL ^−1^) were prepared by making appropriate dilutions in methanol.

Plasma calibration standards and plasma quality control samples were prepared by spiking 10 μL of appropriate standard solutions of LPV in 90 μL of drug-free rat plasma to obtain final concentrations of 250, 500, 1000, 1500, 2000, 2500, 3000, 4000 ng mL^−1^ for calibration curve and 800, 1600 and 3200 ng mL ^−1^ for lower quality control (LQC), medium quality control (MQC) and higher quality control (HQC) samples, respectively. Blank sample was prepared by spiking 10 μL of methanol (drug diluent) in 90 μL of blank plasma. All solutions were stored at 4 °C until further use.

### Extraction Technique

A simple, single-step protein precipitation method was followed for extraction of LPV from Wistar rat plasma. 100 μL of drug spiked plasma sample was pipetted into a RIA vial and 350 μL of acetonitrile (protein precipitating solvent) was added to it and vortex mixed for 2 min. Samples were then centrifuged at 9000 rpm at 4 °C for 20 min. From the centrifuged samples 300 μL of supernatant was transferred to a sample loading vial and injected into the HPLC system.

### Calibration and Calculation Procedures Method Validation

The developed method was validated statistically as per guidelines given by International Conference on Harmonization [33] and United States Pharmacopoeia [34]. Various validation parameters of the developed method were determined using following procedures:

#### Selectivity

**Fig. 2:**
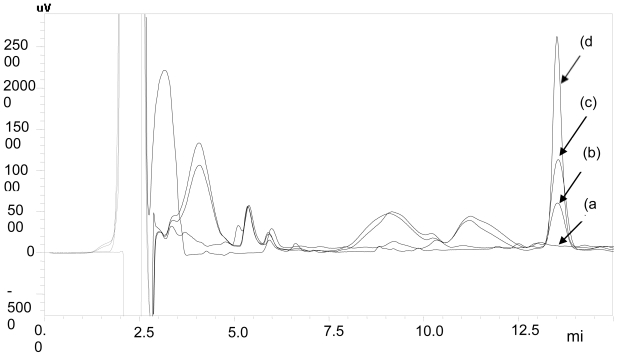
Overlaid chromatograms of (a) blank plasma, (b) *in vivo* test sample, (c) plasma calibration standard (1500 ng mL^−1^) and (d) aqueous standard (3200 ng mL^−1^)

Selectivity of the method can be defined as non-interference by the proteins and other impurities present in the bio-matrix at the retention time shown by LPV. Six different lots of drug-free rat plasma samples were extracted and analyzed for assessment of specificity and selectivity. Overlaid chromatograms of blank plasma, *in vivo* test sample, plasma calibration standard (1500 ng mL^−1^) and aqueous standard (3200 ng mL^−1^) are shown in Fig. 2.

#### Linearity

Plasma calibration standards were prepared and analyzed in five independent runs. Daily standard curves were constructed using the observed peak area to that of nominal concentration. Unknown concentrations were computed from the linear regression equation of the peak area against the concentration. Calibration curve was constructed from a blank sample (plasma sample processed without drug) and eight non-zero concentrations ranging from 250 ng mL^−1^ to 4000 ng mL^−1^. The data is presented in Table 1.

#### Accuracy

For determining the accuracy of the proposed method, different quality control (QC) levels of LPV in plasma (LQC = 800 ng mL^−1^, MQC = 1600 ng mL^−1^ and HQC = 3200 ng mL^−1^) were prepared independently and analyzed (*n* = 6). The data is presented in Table 2.

#### Precision

Repeatability was determined by analyzing all three QC levels of drug concentrations. Inter-day and intra-day variation and analyst variations were studied to determine intermediate precision of the proposed method. Three QC levels of drug concentrations in triplicates were prepared twice in a day and studied for intra-day variation (*n* = 6). The same protocol was followed for three different days to study inter-day variation (*n* = 18). The percent relative standard deviation (%RSD) was calculated from the predicted concentrations obtained by regression equation. The data is presented in Table 3.

#### Sensitivity

Limit of quantification (LOQ) is defined as minimum concentration of LPV in plasma sample that can be quantified with less than 20% RSD [33]. In order to determine LOQ, six independent plasma samples containing 250 ng mL^−1^ of LPV were prepared and analyzed using developed method. The peaks were integrated and concentrations were calculated using calibration equation. Mean concentration and %RSD for these six values were determined.

#### Recovery

Recovery of the drug was determined by comparing the area obtained from plasma (extracted) samples with analytical standard (unextracted) samples. For the recovery experiment, plasma extracted samples were prepared by spiking LPV at three different concentration levels (LQC, MQC and HQC) in triplicate. Precision of LPV recovery at each level (*n* = 3) was determined. Results obtained are presented in Table 4.

#### Stability

**Fig. 3:**
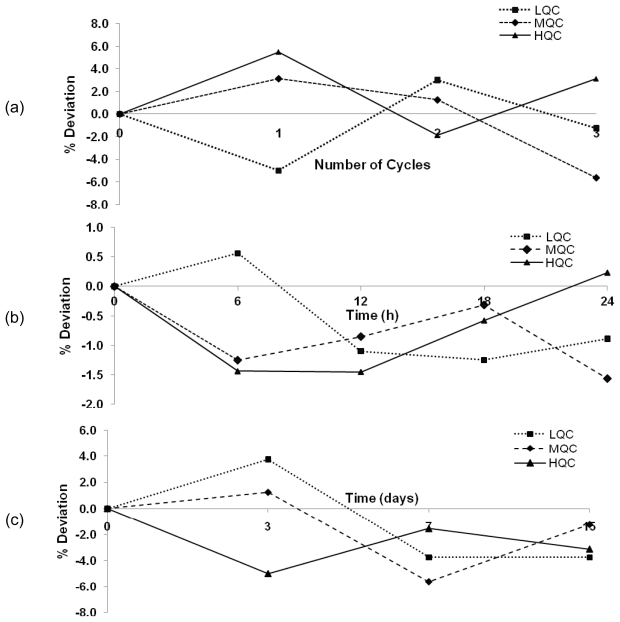
Stability study of LPV in rat plasma (a) Freeze thaw stability; (b) Post extraction stability; (c) Long term stability. Each point represents mean of three independent determinations

Freeze-thaw stability of LPV in rat plasma was determined using three QC (LQC, MQC and HQC) samples for three freeze-thaw cycles. Total of four sets were prepared in triplicates and one set of the prepared concentrations was analyzed on the day of preparation (no freeze-thaw cycle) and the remaining three sets were frozen at −20 °C for 24 h. Frozen samples were thawed by keeping the sealed tubes at room temperature for at least 1 h. One set in triplicate was analyzed and the remaining two sets were kept at −20 °C for freezing and were analyzed after two and three freeze-thaw cycles. The percentage deviation from the mean concentrations observed on day of preparation was calculated and is presented in Fig. 3a.

Long-term stability of LPV in rat plasma was determined by preparing three QC samples (LQC, MQC and HQC). A total of four sets were prepared in triplicates and one set of the prepared concentrations was analyzed on the day of preparation. The remaining three sets were frozen at −20 °C. One set each of stored samples was analyzed after 3, 7 and 15 days of sample preparation by thawing them at room temperature. The percentage deviation from the mean concentrations observed on day of preparation was calculated and the values obtained are shown in Fig. 3b.

Post extraction stability of the processed samples of LPV in rat plasma was investigated by preparing five sets of QC samples (LQC, MQC and HQC) in triplicates. Processed samples were kept in the sample rack of auto sampler and samples were analyzed in triplicates every 6 h for 24 h period on the day of preparation. The percentage deviation from the mean concentrations observed at zero time was calculated. Results obtained are shown in Fig. 3c.

### Pharmacokinetic Study

LPV formulation for intravenous (IV) bolus administration was prepared by dissolving the drug in solvent mixture of ethanol, PEG 400 and water (10:40:50) just before the commencement of study. Formulation was administered through tail vein in male Wistar rats (*n* = 6), weighing 180 to 220 g, at a dose of 5 mg kg^−1^ [35]. Blood samples were drawn from retro-orbital plexus of Wistar rats at 0.083, 0.167, 0.25, 0.5, 1, 2, 3, 4, 6, 8, 12 and 24 h post dose in microfuge tube pretreated with sodium citrate solution (3.8% *w/v*). A baseline blank plasma sample was drawn from each animal before drug administration. All samples were processed according to the procedure described earlier and analyzed using the validated HPLC method.

Various pharmacokinetic parameters were calculated from measured LPV plasma concentrations verses time profiles after IV bolus administration using non-compartmental model and compartmental models in WinNonlin Professional software (Version 4.0, Pharsight Corporation, USA).

## Results and Discussions

### Method Development

Mobile phase consisting of aqueous phase (10 mM ammonium acetate, pH 6.5) and acetonitrile (35:65 *v/v*) at a flow rate of 1 mL min^−1^ was selected as optimal condition for the developed method. With optimized mobile phase condition, retention time of LPV was found to be 13.5 ± 0.15 min with an asymmetric factor of 1.21 ± 0.10. Retention time of LPV was increased to 21.02 min with decrease in acetonitrile composition from 65% to 50% *v/v* and showed diminished height, whereas the same composition of methanol (65% *v/v*) showed peak broadening and increased retention time. It was found that increase in pH decreases the retention time of LPV. Sharp and symmetrical peak shape with reasonable retention time was observed in range of pH 6.5 to 7.0. It was observed that 10 mM ammonium acetate provided a pH value of 6.5. Hence, it was chosen as buffer without further alteration in the composition.

### Method Validation

#### Selectivity

Simple and efficient one-step precipitation technique was found to be suitable for estimation of LPV in rat plasma. No interference was observed from endogenous protein impurities in processed test samples as well as blank plasma sample at retention time of the drug as shown in Fig. 2. Thus, the proposed method was found to be specific and selective for the estimation of LPV in rat plasma.

#### Linearity

Different concentrations and their corresponding areas are shown in Table 1. At all the concentration levels, %RSD did not exceed 5.93. The plasma calibration curve was linear over the calibration range of 250 ng mL^−1^ to 4000 ng mL^−1^. According to linear regression analysis, the slope (± standard error) and intercept (± standard error) were found to be 90.34 (± 0.87) and 2475.66 (± 532.97), respectively, with a regression coefficient value of 0.996. Lower values of standard error of estimate (5.63) and MSSR (1.95 × 10 ^−4^) indicates high precision of the developed method. Lower *Fcal* value of 0.039 in comparison to *Fcrit* (5,35) value of 2.48 at *P* < 0.05, further confirmed precision of the proposed method.

#### Accuracy

All three quality control samples (LQC = 800 ng mL^−1^, MQC = 1600 ng mL^−1^ and HQC = 3200 ng mL^−1^) showed an accuracy ranging from −1.37% to 1.47% with maximum %RSD of 4.78 across all the QC levels, establishing the accuracy of method for LPV estimation in rat plasma (Table 2).

#### Precision

In a repeatability study, %RSD ranged from 0.32 to 4.96 across all QC samples (Table 3). The %RSD values for intra-day variation were not more than 4.96 and for inter-day variation were less than 4.73 (Table 3). Acceptable %RSD values indicated the repeatability and intermediate precision of the method.

#### Sensitivity

The mean percentage accuracy of six independent samples of 250 ng mL^−1^, calculated against calibration equation and was found to be 93.1 with %RSD value of 4.88. Hence, the concentration of 250 ng mL^−1^ was considered as lowest limit of quantification (LLOQ) for the proposed method.

#### Recovery

The absolute recovery of LPV from the spiked rat plasma samples, when compared with analytical standards of same concentration, was within 97.5% to 100.19% with %RSD less than 4.03 at each concentration levels. The high (nearly 100%) mean percent recovery values (Table 4) which precludes the use of internal standard and low %RSD values (%RSD < 5.0) established the extraction efficiency of the selected solvent for precipitation and also robustness of the method.

In the HPLC methods reported by Usami *et al*. and Faux *et al.* for estimation of LPV in human plasma, sample preparation involved liquid-liquid extraction and solid-phase extraction methods, respectively [32, 33]. Such sample preparation methods involve many processing steps which are complex and time-consuming and require costly solvents/solid-phase extraction cartridges. Internal standard was also used in both the methods. Based on the reported methods, plasma sample of at least 500 μL is required in sample preparation. To obtain 500 μL of plasma a minimum of 1 mL blood is required to drawn, at each time point, from subjects involved in the *in vivo* study. Therefore the reported methods are not suitable in preclinical pharmacokinetic evaluation of formulations containing LPV.

#### Stability

The stability of LPV in rat plasma was evaluated using QC samples under different stress conditions and the results obtained are shown in Fig. 3. In freeze-thaw stability, no significant degradation of LPV was observed up to three cycles over a period of three days. The deviation from the zero time concentration was found to be less than 8.0% at the end of three freeze-thaw cycles as shown in Fig. 3a. In post extraction stability study of the processed samples, LPV was found to be stable for 24 h, with a maximum deviation of less than 2.0% from the zero time concentration as shown in Fig. 3b

In long-term stability studies, LPV was found to be stable for 15 days when stored at −20 °C. The deviation in recoveries of LPV after analysis at 3, 7 and 15 days of sample preparation was found to be within acceptable limits (Fig. 3c). The results of this study indicated that storage temperature of −20 °C was adequate for storing the samples for at least 15 days.

### Pharmacokinetic Application

**Fig. 4:**
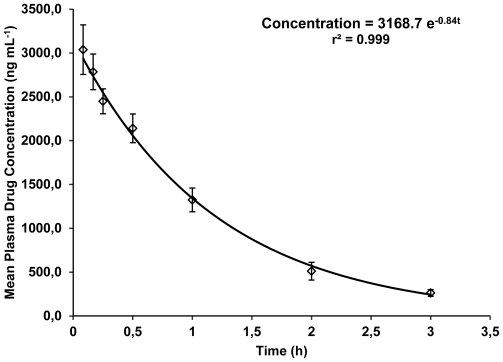
The mean plasma concentration versus time profile of LPV in rats after intravenous bolus administration of the drug (5 mg kg^−1^, *n* = 6)

The developed and validated HPLC method was applied to determine the pharmacokinetic parameters following IV bolus administration of LPV formulation in rats. The mean plasma concentration versus time profile of LPV obtained following the IV bolus administration is given in Fig. 4.

The time course of plasma drug concentration was found to follow a mono-exponential equation, Concentration = 3168.7 e^−0.84t^, with a good correlation coefficient (r^2^ = 0.999) indicating that the drug follows one compartment open model in rats. The pharmacokinetic parameters obtained from the study using non-compartmental and compartmental analysis were area under the curve (AUC) = 3850.33 ± 263.87 h ng mL^−1^, area under the first-moment curve (AUMC) = 4403.07 ± 171.33 h^2^ ng mL^−1^, mean retention time (MRT) = 1.15 ± 0.05 h, concentration at time zero (Co) = 3168.68 ± 289.88 ng mL^−1^, elimination half-life (t½) = 0.82 ± 0.03 h, volume of distribution (Vss) = 1592.09 ± 146.44 mL kg^−1^ and total plasma clearance (CLs) = 1344.79 ± 4.40 h^−1^ mL kg^−1^.

Samples collected till 3 h (approximately equal to 4 times half-life of drug) post IV bolus administration of the drug were analyzed in the study indicating the sensitivity and applicability of the proposed HPLC method in pharmacokinetic studies of the drug in rats.

## Conclusions

The developed and validated HPLC method for estimation of LPV in Wistar rat plasma was found to be rapid, precise, specific, reproducible and cost-effective. Recovery of LPV from plasma samples by protein precipitation technique using acetonitrile was found to be efficient. In addition, the drug was found to be stable under various processing and storage conditions. The method allows high sample throughput due to simple procedure for sample preparation and relatively short run time. The method was successfully employed in determining the pharmacokinetic parameters of the drug following IV bolus administration in rats.

## Figures and Tables

**Tab. 1:** Calibration data of LPV in Wistar rat plasma

Concentration (ng mL^−1^)	Mean area^a^ (± SD^b^)	% RSD^c^	% Mean recovery d (± SD^b^)
250	23612.5 ± 1400.78	5.93	100.32 ± 3.91
500	48157.0 ± 660.44	1.37	99.89 ± 2.32
1000	100514.5 ± 3517.86	3.50	99.19 ± 1.44
1500	130077.0 ± 2839.74	2.18	98.92 ± 1.08
2000	186722.5 ± 4951.87	2.65	99.50 ± 1.32
2500	231054.0 ± 9192.39	3.98	99.19 ± 1.11
3000	266898.0 ± 9779.29	3.66	98.96 ± 1.02
4000	367433.5 ± 1402.19	0.38	98.95 ± 0.94

a Each value is mean of five independent determinations (*n* = 5);

b Standard deviation;

c Percentage relative standard deviation;

d Percent drug recovery = [(Peak area of plasma standard/peak area of analytical standard of same concentration) ×100].

**Tab. 2:** Accuracy and precision data for the proposed method in Wistar rat plasma

Level	Predicted concentration^a^ (ng mL^−1^)			Mean accuracy^e^ (%)
	Range	Mean^b^ (± SD^c^)	%RSD^d^	
LQC (800 ng mL^−1^)	737–842	789.00 ± 37.74	4.78	−1.37
MQC (1600 ng mL^−1^)	1608–1640	1623.46 ± 12.17	0.75	1.47
HQC (3200 ng mL^−1^)	3168–3247	3219.54 ± 27.72	0.86	0.61

a Each value is mean of six independent determinations (*n* = 6);

b Predicted concentration of LPV was calculated by linear regression equation;

c Standard deviation;

d Percentage relative standard deviation;

e Accuracy is given in relative error % =[100 × (predicted concentration – nominal concentration)/nominal concentration)].

**Tab. 3:** Results of intermediate precision study in Wistar rat plasma

Level	Intra-day repeatability (%RSD^a^) (*n* = 3)			Inter-day repeatability (%RSD^a^) (*n* = 18)
	Day-1	Day-2	Day-3	
LQC	4.37	3.30	4.34	4.73
	4.43	4.21	4.96	
MQC	0.32	2.17	1.89	1.44
	0.43	1.30	0.63	
HQC	1.03	0.69	0.60	1.57
	0.57	0.46	0.80	

a Percentage relative standard deviation.

**Tab. 4:** Absolute recovery of LPV from plasma samples following protein precipitation extraction method

Nominal concentrations (ng mL^−1^)	% Mean recovery^a^ (± SD^b^)	%RSD^c^
800 (LQC)	99.32 ± 4.01	4.03
1600 (MQC)	97.50 ± 1.32	1.35
3200 (HQC)	100.19 ± 1.44	1.44

a Percent drug recovery = [(Peak area of plasma standard/peak area of analytical standard of same concentration) ×100];

b Standard deviation;

c Percentage relative standard deviation.
